# Developing a Clinical Service in Interventional Radiology: Results from the 2024 CIRSE Clinical Practice Survey

**DOI:** 10.1007/s00270-024-03858-y

**Published:** 2024-10-09

**Authors:** A. G. Ryan, B. Slijepčević, A. Cannavale, M. Krokidis, J. Y. Chun, T. de Baere, R. Dezman, S. Duvnjak, M. A. Ruffino, J. Urbano, A. H. Mahnken

**Affiliations:** 1 University Hospital Waterford and Royal College of Surgeons in Ireland, Waterford, Ireland; 2 Cardiovascular and Interventional Radiological Society of Europe, Vienna, Austria; 3 Department of Radiological Sciences, Policlinico Umberto I University Hospital, Rome, Italy; 4 1St Department of Radiology, Medical School, Areteion University Hospital, National and Kapodistrian University of Athens, Athens, Greece; 5 Department of Diagnostic, Interventional and Pediatric Radiology (DIPR), Inselspital, Bern University Hospital, University of Bern, Bern, Switzerland; 6 George’s University Hospitals NHS Foundation Trust, London, UK; 7 Radiologie Interventionnelle, Institut Gustave Roussy, Villejuif, France; 8 Clinical Institute of Radiology, University Medical Centre Ljubljana, Ljubljana, Slovenia; 9 Department of Vascular Surgery, Rigshospitelet, Copenhagen, Denmark; 10 Interventional Radiology, Institute of Imaging of Southern Switzerland - EOC Lugano, Lugano, Switzerland; 11 Vascular and Interventional Radiology Department, Hospital Universitario Ramón Y Cajal, Madrid, Spain; 12 Department of Diagnostic and Interventional Radiology, University Hospital Marburg, Philipps-University Marburg, Baldingerstrasse, 35043 Marburg, Germany

**Keywords:** Interventional radiology, Clinical practice, Quality standards, Patient care, Practice development, Clinical practice

## Abstract

**Purpose:**

Engaging in clinical service development is a prerequisite for Interventional Radiology (IR) to prosper as a full clinical discipline. The CIRSE Clinical Services in IR Task Force conducted a survey of CIRSE members worldwide to assess the current status of their clinical practice and to identify areas of practice requiring further support.

**Materials and Methods:**

An online questionnaire with 63 structured items was sent to 7,501 CIRSE members in November 2023. The survey was closed in January 2024 and a statistical data analysis was performed.

**Results:**

A total of 520 complete responses were collected. 49.6% of respondents have an IR outpatient clinic, 34.5% have a dedicated IR day-case ward and 19.8% have dedicated inpatient beds. While 62% of respondents treat patients as the primary consultant responsible for their patients’ care, 40.3% of respondents currently without their own beds have admitting rights to the hospital. Clinical practice activities are itemised in the work schedule of 41.3% of respondents and 45% routinely perform ward rounds. A total of 40% feel very positive with their personal clinical practice competency.

**Conclusion:**

With half of responding IRs having primary patient access and clinical services in place, the results are encouraging; however further engagement by those who are not yet involved is required. The authors advocate a step-wise approach towards clinical services starting with ward rounds, and subsequently taking increasing responsibility for each step in the IR patient pathway.

**Graphical Abstract:**

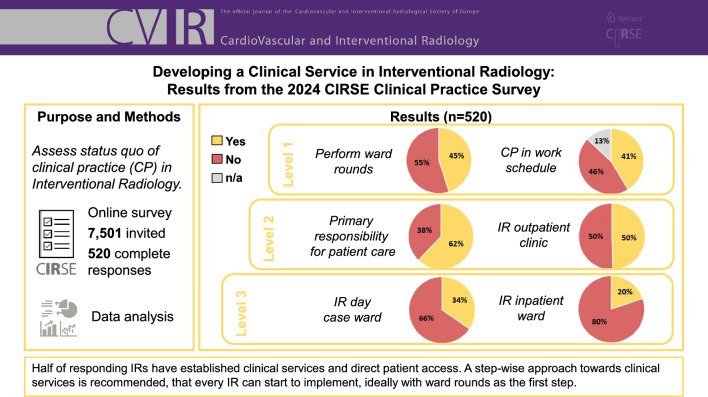

## Introduction

Interventional radiology (IR) is evolving towards an independent clinical discipline, and this process represents a major priority for CIRSE under its new vision for the future of IR [1]. To support this, a dedicated task force was established, one of its first duties being to establish the current baseline of clinical practice with a member survey.

## Materials and Methods

The authors developed a questionnaire with 63 structured items. Most questions were single choice followed by open-response fields with logic-based display of follow-up questions. The survey was set up in an online tool (Alchemer LLC, USA) and sent to CIRSE members worldwide (7,501) on November 29, 2023, followed by two reminders. Data collection was closed on January 19, 2024. A descriptive statistical analysis was performed in Microsoft Excel (2016, Microsoft Corporation, USA). Only complete responses were included in the analysis.

## Results

A total of 520 complete responses were received, corresponding to a response rate of 7% (above average compared to other recent CIRSE surveys). A majority (57.5%) of respondents were European-based, the largest groups of responders working in the UK (9.8%), Germany (9.6%), Italy (8.7%), and Spain (7.5%). Exactly 50% of respondents were self-declared “full-time IRs”, dedicating 80–100% of their workload to IR. Another 18% indicated dedicating 60–80% of their time to IR. A majority of respondents work in academic/university hospitals (56%) or general hospitals (public, 22%), with a clear trend towards large centres with over 800 beds (45%) or 400–799 beds (28%).

**Fig. 1:**
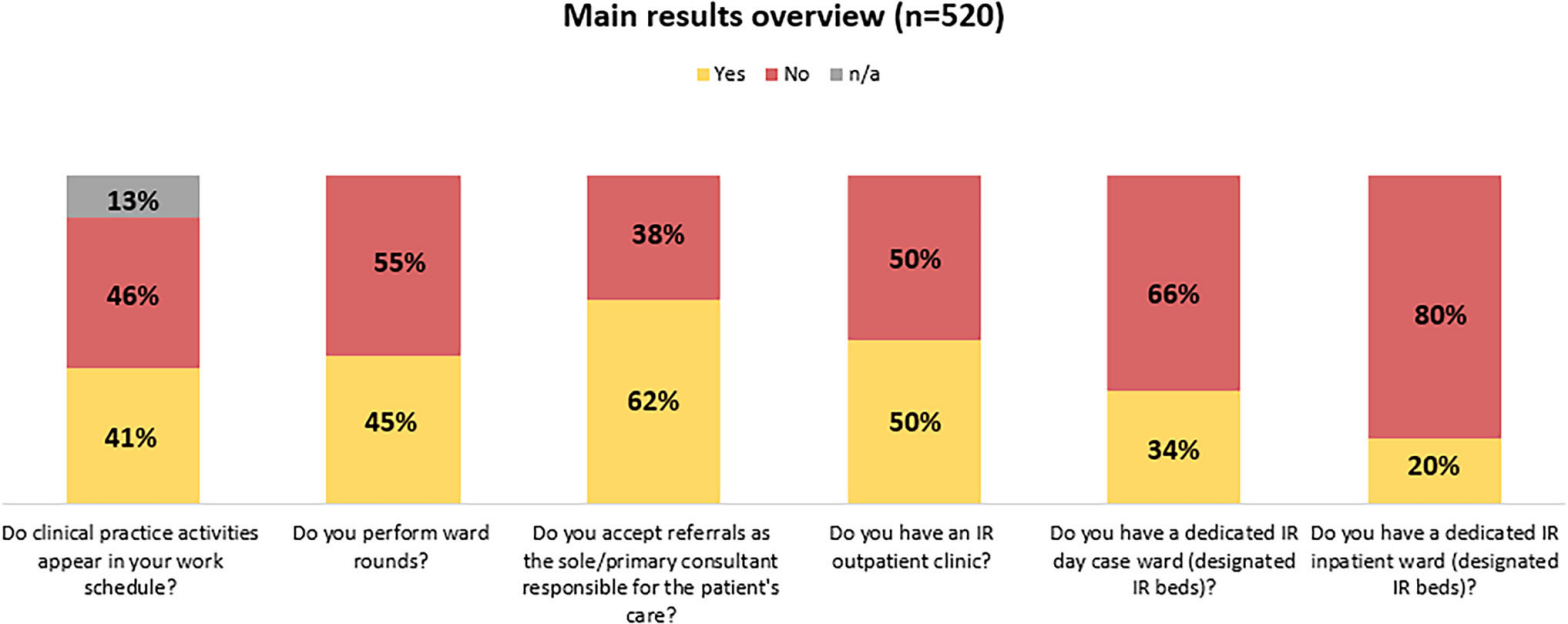
Overview of main results

Among all respondents, 62.1% accept patients as the sole or primary consultant for the patient’s care and 52.7% indicate taking primary responsibility for their patients’ treatment plans, involving other specialties only on an ‘as required’ basis. Ward rounds are performed by 45% of respondents, with 95.7% of this subsample (n = 234) performing post-procedural ward rounds on their patients and 73.9% performing pre-procedural ward rounds on patients referred to them by other services (Fig. 1). For 41.3% of respondents, clinical practice activities such as ward rounds or outpatient clinics explicitly appear in their work schedule. When asked which activities appear in the work schedule (multiple choice), outpatient consultations (93%), clinical rounding (63.7%), and Multidisciplinary Team Meetings (MDT) (61.9%) were most frequently cited. In 90.2%, informed consent for IR procedures is taken by a member of the IR/Radiology team, and 73.3% independently write prescriptions, e.g., for drugs/medicinal products (89.9%) and diagnostic tests (89.3%). Continuous quality improvement activities dedicated to IR are performed by 68.1%, including morbidity and mortality meetings (70.9%), daily case discussions (68.1%), and MDT outcome (56.5%, multiple answers possible). Patient safety checklists are rountinely used by 77.3%.

In terms of access to beds and infrastructure, 49.6% of respondents have an IR outpatient clinic, which is located in most cases in a room in the Radiology/IR department (57.4%), in the same outpatient department as other services in the hospital (31.8%), or in an imaging room (14.3%; multiple responses possible). 34.5% of respondents have a dedicated IR day-case ward (designated IR beds), and 19.8% reported having dedicated inpatient beds (Fig. 1).

Among respondents without a dedicated inpatient ward (n = 417), 40.3% have admitting rights to their hospital. Of these, 82.7% and 95.2% can admit inpatients and outpatients respectively. For those admitting patients without a dedicated IR ward, patients are mostly admitted to beds located in a surgical (77.7%) or medical ward (69.9%), followed by obstetrics and gynaecology (27%) and other (28.4%). Among all respondents, 78.7% admit patients through the service of other clinicians. Among those IRs without a dedicated day-case ward (n = 340), 60.6% still treat outpatients. The majority routinely sees their patients in the outpatient clinic or the IR’s office before (70%) and after (63.8%) the procedure. A majority of respondents indicated having IR trainees and fellows (67.9%), dedicated non-physician staff for IR (88.7%), and dedicated IR clerical staff (58.5%) in their departments.

Reasons why no IR clinical service has been developed (n = 284) include IR procedural workload (20.6%), diagnostic radiology workload (20.6%), opposition from hospital management (14.6%) or hospitals refusing access to beds (13.3%), opposition from group colleagues (9.6%) or no outpatient clinic space (8.7%). Only 2.5% indicated they did not believe a clinical service is necessary.

**Fig. 2:**
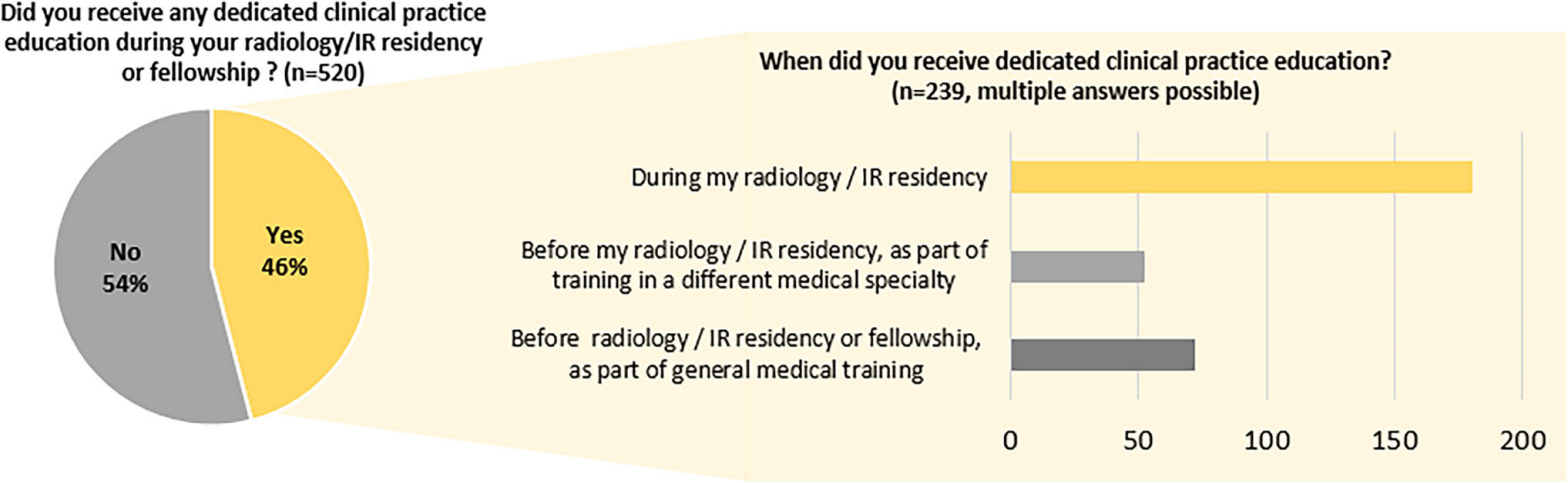
Clinical practice training

A total of 46% of respondents received dedicated clinical practice education during their Radiology and/or IR residency or fellowship (Fig. 2). When asked about their personal clinical practice competency, 40% feel ‘very confident’, 35% feel ‘rather confident’, 14% are ‘neutral’ and 11% are ‘not’, or ‘not very’ confident and 54% are satisfied with their current level of clinical practice.

## Discussion

For a sustainable IR practice, IRs need to assume primary responsibility for managing the patient and their condition [2]. Respondents confirm almost unanimously (97.5%) that developing a clinical service in IR is necessary, regardless of their current level of practice or roadblocks in place at present. Patient satisfaction is increased by IRs taking responsibility for the entire patient pathway [3].

**Fig. 3:**
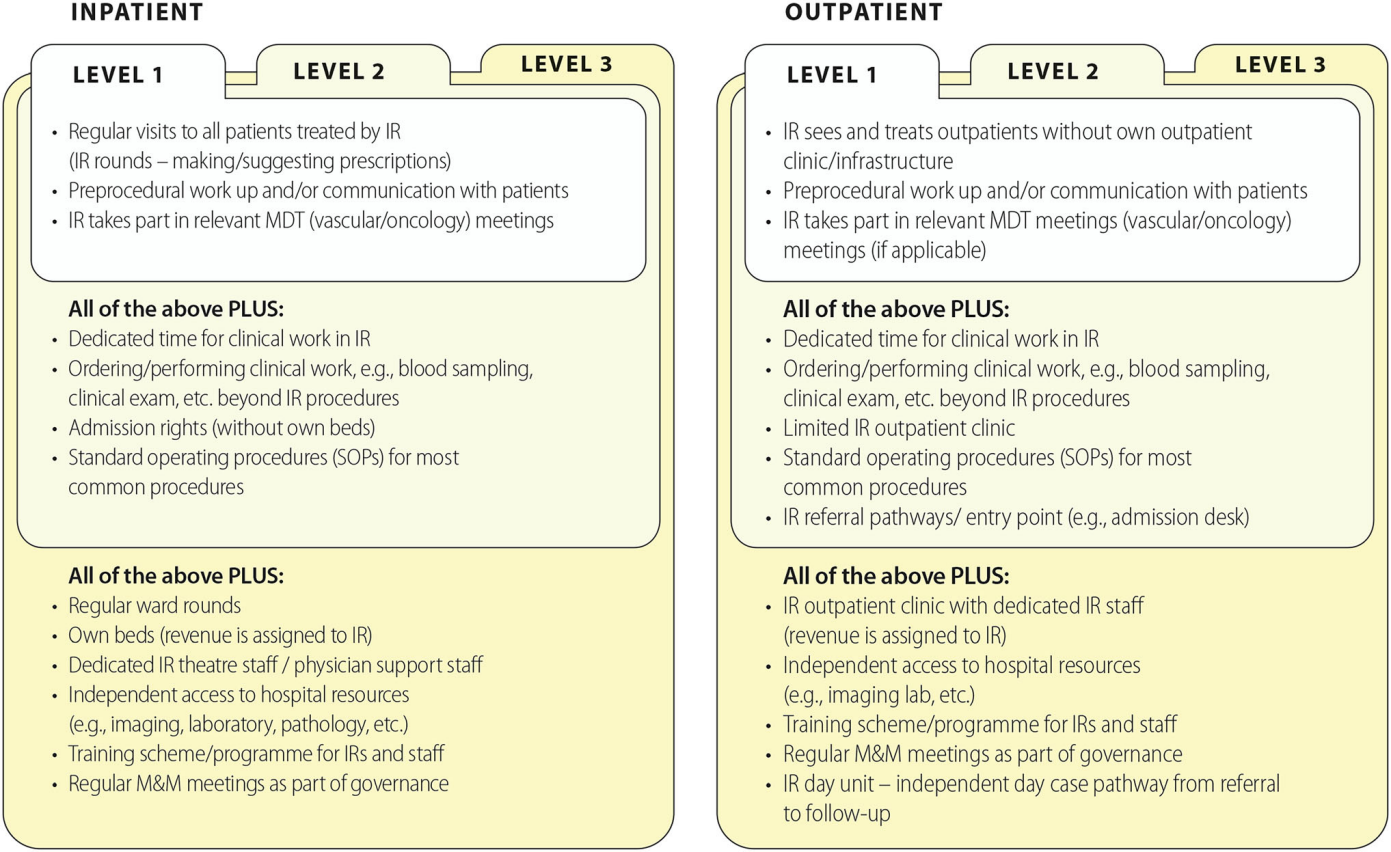
CIRSE levels of clinical services infrastructure

While work realities vary greatly for IRs depending on their location [4], there are many ways to start a clinical service. The task force has developed three levels of clinical services infrastructure in IR (Fig. 3), which can serve as a benchmark for their current level of clinical involvement, and as a template to continue developing clinical services. The authors propose that the first and most important step is to increase the levels of communication with patients beyond the procedural setting, e.g. by performing pre- and post-procedural ward rounds. Although a reasonably high percentage of respondents see their patients in the outpatient setting before and after the procedure, only 45% answered yes to the question “Do you perform ward rounds?”. This situation should be addressed, as ward rounds afford a better assessment of patients’ needs, and provide an opportunity to ask questions, thereby building a rapport and optimising patient care [5]. Positively, of the subsample performing ward rounds (n = 234), 95.7% perform post-procedural ward rounds on their patients and 73.9% perform pre-procedural ward rounds.

When performing rounds, it is imperative that they be documented in the patient’s chart or the Electronic Patient Record, not just for clinical purposes, but also to increase IRs’ visibility. Increased visibility among referring physicians is also achieved by regular participation in multidisciplinary team meetings as an IR, independent from the chairing diagnostic radiologist. Ideally, dedicated time for such clinical practice activities should appear explicitly on the IR’s work schedule.

The next step towards a full clinical service is for IRs to incorporate more aspects of longitudinal care, starting with an outpatient clinic (which may be in the IR’s office at the outset), and working towards a day ward and ambulatory care. This is a logical progression, as more than half of respondents accept referrals as the primary consultant responsible for patient care or take primary responsibility for their patients’ treatment plans, consulting other clinicians when required, as would a surgeon.

One of the main advantages of IR is the ability to deliver ambulatory care (AC) [5, 6], which has clear advantages in terms of cost and efficiency, in addition to improved patient safety and psychological benefits. For AC to be safe and effective, allocation of adequate resources is necessary [7] which can be a major obstacle initially, especially in countries where inpatient care receives higher reimbursement [8].

The survey showed that 40.3% currently have admitting rights to non-IR beds, through other clinical services, while only one fifth of respondents have access to their own inpatient beds. The authors believe that IRs should work towards having their own inpatient beds; as argued by Bryant et al. [9], admitting rights for IR follow the rationale that the team with best knowledge about procedural and postprocedural issues should be admitting their own patients. While having one’s own beds incurs challenges such as 24/7 IR staffing and dedicated support staff, advantages include improved patient safety and experience, staff education, and overall efficiency [9]. Having responsibility for billing and resources, in combination with documentation of patient and financial outcomes, will contribute to the future visibility and growth of IR, including an increased provision of ‘protected’ IR beds.

In terms of developing one’s clinical practice, the authors recommend that less resource-dependent activities be introduced first and then successively enhanced (Fig. 3). To ensure high quality of provided services, continuing quality improvement measures in IR and the standard use of patient safety checklists are mandatory [10].

Finally, the survey showed that IRs were confident in their clinical performance (75%), but reported low levels of clinical practice education (less than 50% of IRs receiving specific training). Although this figure likely reflects the spread of respondents’ experience (including those who historically developed their clinical practice training in other specialities prior to entering IR), it is imperative that all current and future trainees receive dedicated clinical practice education during their IR training. It is evident that there is a strong need to provide dedicated training opportunities/tools for IRs and thus CIRSE is taking further steps to support, train, and encourage IRs to get more involved, for the benefit of their patients and the sustainability of IR.

Limitations: A 7% response rate and a preponderance of respondents from large academic centres limits the extrapolation of these findings to all IR practices, and, given a presumed self-selection bias of IRs interested in clinical work, the true figures regarding the penetration of clinical practice may be significantly less.

## Conclusion

Overall, the survey results are positive, with half of the responding IRs having established clinical services and patient access. The authors advocate for a step-wise approach towards the development of clinical services that every IR can start to implement at each stage of the IR patient pathway [2], irrespective of their local circumstances. Performing ward rounds is an ideal starting point, and the time to start is now.
